# Bandage Contact Lens-Assisted Plaque Brachytherapy for Anterior Segment Tumors: A Novel Technique to Prevent Corneal Complications

**DOI:** 10.7759/cureus.87727

**Published:** 2025-07-11

**Authors:** Neiwete Lomi, Deepsekhar Das, Bhavna Chawla, Tapashree Ghosh, Dhanabalan Rajasekaran, Radhika Tandon

**Affiliations:** 1 Ophthalmology, All India Institute of Medical Sciences, New Delhi, New Delhi, IND; 2 Medical Physics, All India Institute of Medical Sciences, New Delhi, New Delhi, IND

**Keywords:** anterior segment tumor, bandage contact lens, eye plaque brachytherapy, intraocular tumor, iridociliary melanoma

## Abstract

Anterior segment tumors pose challenges in treatment due to their location and potential for globe loss. Plaque brachytherapy allows globe salvage, but placing plaques over the cornea and limbus may lead to epithelial defects and ulcers. We report a novel technique using a bandage contact lens as a buffer between the plaque and cornea in a 48-year-old woman with iridociliary melanoma. The lens thickness (0.07 mm) was included in radiation dose planning. The patient had no corneal complications and showed tumor regression at seven months. Two additional patients treated similarly showed stable tumors and no adverse effects at six months. This method may offer a safer alternative to amniotic membrane grafts for anterior segment brachytherapy, reducing corneal toxicity without compromising tumor control.

## Introduction

Tumors of the anterior segment of the eye, including iris, iridociliary body, and corneal or conjunctival malignancies, represent a small but clinically significant portion of intraocular and ocular surface neoplasms. Their location in the anterior chamber, often in close proximity to delicate structures such as the cornea, lens, and angle, presents unique challenges in management. Enucleation has traditionally been the treatment of choice for extensive anterior segment tumors, particularly when there is concern for extraocular extension or vision-threatening complications [1]. However, with earlier detection and advances in ocular oncology, globe-sparing therapies have increasingly become feasible for small- to medium-sized lesions [1-3].

Plaque brachytherapy has emerged as an effective treatment modality for selected intraocular tumors, including those involving the anterior segment. This technique involves placing a radioactive source, commonly an iodine-125 or ruthenium-106 plaque, in close proximity to the tumor to deliver localized radiation while minimizing systemic exposure [4]. While it is well-established for posterior segment tumors such as choroidal melanoma, its application in anterior segment tumors requires significant technical modification. Plaques for anterior lesions are often sutured over the cornea and limbus, which differs markedly from their placement on the sclera in posterior segment treatments [5,6].

One of the significant drawbacks of anterior plaque placement is the high risk of ocular surface complications. The direct contact of the plaque with the cornea may lead to persistent epithelial defects, corneal edema, keratitis, and, in some cases, ulceration. The pressure, friction, and radiation exposure to the epithelium contribute to this complication profile. In a study by Semenova et al., up to 41.3% of patients undergoing epicorneal plaque brachytherapy with amniotic membrane grafts (AMGs) developed corneal complications, including epithelial breakdown and edema [7]. While AMGs provide some degree of surface protection, their mechanical durability during plaque application is limited, and they may degrade or shift over time.

To mitigate such complications, the present report introduces a novel technique: the interposition of a bandage contact lens (BCL) between the radioactive plaque and the corneal surface. BCLs are thin, transparent, and biocompatible devices commonly used in clinical ophthalmology to promote epithelial healing, reduce pain, and protect the cornea from mechanical trauma [8]. With a thickness of approximately 0.07 mm, these lenses offer a simple, cost-effective buffer without interfering with surgical visualization or significantly altering plaque dosimetry when accounted for during radiation planning.

This case report describes the successful use of BCL-assisted plaque brachytherapy in a patient with iridociliary melanoma, with no corneal complications observed over a seven-month follow-up period. The technique was further applied in two additional patients with favorable outcomes. We propose this method as a safer alternative to AMGs for anterior segment brachytherapy, particularly when epicorneal plaque placement is necessary.

## Case presentation

A 48-year-old female presented with a six-month history of painless diminution of vision in the right eye. She also reported noticing a pigmented area in the superior part of the eye. There was no history of trauma, systemic malignancy, or ocular surgery. On examination, her best-corrected visual acuity was 6/60 in the right eye and 6/6 in the left eye. Intraocular pressure was 14 mm Hg in both eyes.

**Figure 1:**
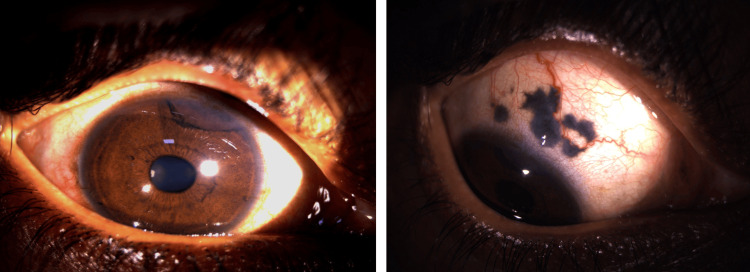
Slit-lamp image of the right eye showing a pigmented lesion on the superior iris, along with pigmentation of the superior sclera

Anterior segment examination of the right eye revealed a pigmented lesion extending from the 12 o’clock to 2 o’clock position on the superior iris. Adjacent scleral pigmentation was also noted in the corresponding superior quadrant. The lesion appeared to extend posteriorly, raising suspicion for involvement of the ciliary body. The left eye examination was unremarkable (Figure 1).

**Figure 2:**
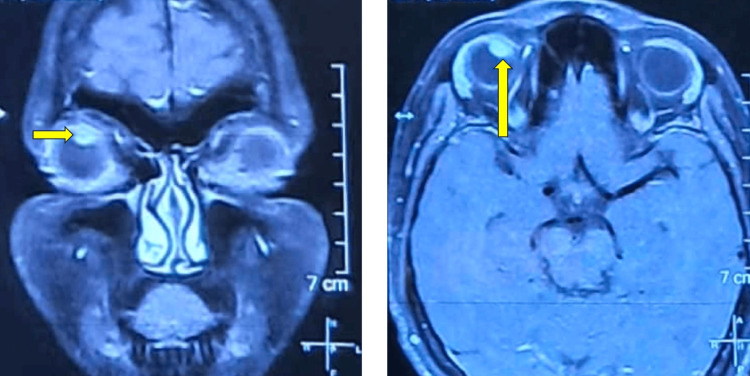
CEMRI of the brain and orbit showing coronal and axial cuts with a hyperintense lesion in the superior aspect of the right orbit a: Coronal section of CEMRI showing a hyperintense lesion in the superior quadrant of the right eye highlighted with a yellow arrow. b: Axial section of CEMRI showing a hyperintense lesion in the right eye highlighted with a yellow arrow. CEMRI: contrast-enhanced magnetic resonance imaging

**Figure 3:**
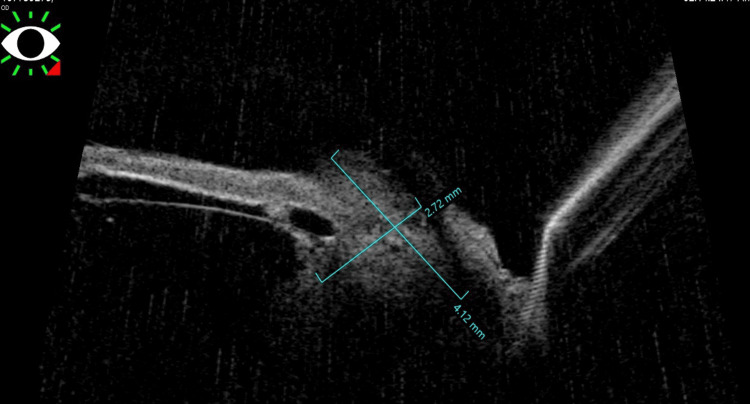
UBM image showing an iridociliary lesion measuring 2.72 × 4.12 cm UBM: ultrasound biomicroscopy

Ultrasound biomicroscopy (UBM) of the right eye confirmed the lesion to be iridociliary in origin. The mass measured approximately 2.75 mm in thickness and 4.12 mm in basal diameter (Figure 2). A contrast-enhanced MRI scan of the orbit was performed for further evaluation. The lesion appeared hyperintense on T1-weighted images and hypointense on T2-weighted images, consistent with the radiologic characteristics of melanoma (Figure 3). The lesion was localized to the iridociliary apparatus, with no evidence of extrascleral extension or orbital involvement.

**Figure 4:**
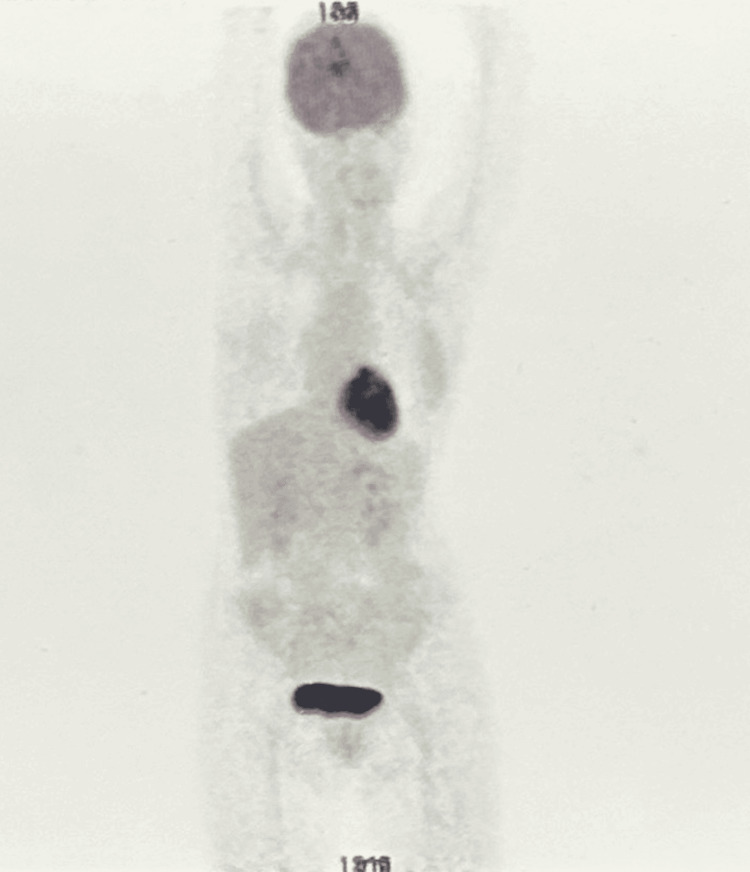
Whole-body PET scan showing no obvious FDG-avid lesions, thereby ruling out metastasis PET: positron emission tomography, FDG: fluorodeoxyglucose

Based on the clinical, imaging, and UBM findings, a diagnosis of iridociliary melanoma was made. A PET scan was performed to rule out metastasis (Figure 4). After a multidisciplinary discussion and patient counseling regarding treatment options, the decision was made to proceed with globe-salvaging plaque brachytherapy using ruthenium-106 (Video 1).

**Video 1:** BCL-assisted plaque brachytherapy

Treatment planning included the use of a P01 applicator, with a physical outer diameter of 12 mm and an active core diameter of 9.55 mm. A total radiation dose of 80 gray (Gy) was prescribed to a depth of 3.7 mm from the plaque surface, with a scleral surface dose of 244 Gy. The calculated duration of radiation delivery was 91 hours.

The patient underwent surgery under local anesthesia. A superior conjunctival peritomy was performed to expose the pigmented scleral region. Under direct visualization, a dummy plaque was positioned to confirm appropriate coverage of the iris and adjacent scleral lesion. Once accurate placement was confirmed, the dummy plaque was replaced with the radioactive plaque, which was sutured in place using 5-0 Ethibond sutures.

To minimize mechanical trauma and radiation-induced corneal epithelial damage, a BCL was inserted between the radioactive plaque and the corneal surface. The contact lens acted as a buffer, reducing direct plaque-corneal contact. A temporary tarsorrhaphy was then performed using 4-0 silk sutures to maintain plaque stability and minimize eyelid movement.

After 91 hours, the plaque was removed. Throughout the treatment period, the plaque remained well-positioned with no evidence of displacement. Notably, there were no signs of corneal epithelial defects, ulceration, or discomfort. The patient tolerated the procedure well, and no complications were observed postoperatively.

## Discussion

Anterior segment tumors such as iridociliary melanoma present a therapeutic challenge due to their proximity to critical ocular structures and their potential for local invasion. Globe-sparing treatment options, particularly plaque brachytherapy, are effective for small to medium-sized tumors but require technical adaptations when lesions involve the anterior segment. Unlike posterior uveal melanomas, where the plaque is secured over the sclera, anterior segment tumors necessitate plaque placement over the cornea or limbus. This positioning increases the risk of radiation-induced or mechanical corneal complications, including epitheliopathy, edema, and ulceration [1,2].

Various strategies have been developed to reduce these complications, the most common being the use of AMGs. AMG has anti-inflammatory, anti-scarring, and epithelial-promoting properties, making it a valuable adjunct in ocular surface reconstruction [1]. Semenova et al. reported that despite using AMG during epicorneal plaque placement, 41.3% of patients developed corneal complications, indicating that while AMG may offer biological benefits, it does not provide robust mechanical protection [7]. The biologic nature of AMG also poses limitations: it may degrade during the plaque treatment period, shift from its intended position, or fail to uniformly cover the corneal surface, especially in mobile or blinking eyes.

In contrast, BCLs offer a mechanical barrier that remains stable throughout plaque placement. These lenses are designed to protect the corneal epithelium from friction and shear forces, promote epithelial healing, and reduce patient discomfort [8]. In our case, a BCL of 0.07 mm thickness was used; its presence was accounted for during radiation dosimetry planning. The BCL created a uniform interface between the radioactive plaque and the corneal epithelium, ensuring that no part of the plaque directly abutted the ocular surface. Over a 91-hour treatment period, the patient exhibited no epithelial defect, discomfort, or signs of corneal toxicity. This finding was replicated in two additional cases, all of which demonstrated plaque stability and intact corneal surfaces at follow-up.

BCLs also offer practical advantages. They are inexpensive, easy to apply, widely available, and do not require special storage or preparation. Unlike AMG, they do not involve biologic material, reducing the risk of immunologic reactions or graft failure. Additionally, BCLs allow for better control of plaque-to-corneal contact geometry, which can be consistently accounted for in treatment planning. These features make BCLs a particularly attractive alternative to AMG for plaque protection in anterior segment tumors [8].

Although early results are promising, limitations include the small number of patients and short follow-up duration. Long-term studies comparing BCLs and AMG directly in controlled settings are needed to validate their relative efficacy. However, this case series supports the use of BCLs as a safer, more stable mechanical buffer during anterior segment plaque brachytherapy.

## Conclusions

Anterior segment plaque brachytherapy presents unique challenges due to the need for epicorneal plaque placement, which poses a risk of corneal epithelial damage. This case highlights a novel and practical approach using a BCL as a mechanical barrier between the radioactive plaque and the corneal surface. Compared to AMGs, BCLs offer consistent, stable protection without degradation or displacement over time. In our experience, this technique effectively preserved corneal integrity and provided excellent plaque stability without compromising tumor control. BCL-assisted plaque placement may serve as a simple, safe, and effective alternative for corneal protection in anterior segment radiotherapy. Further studies with larger cohorts and long-term follow-up are warranted to validate these findings and to establish standardized guidelines for this technique.

## References

[1] Pike S, Engelhard SB, Greig LC, Woods K, Jennelle RL, Berry JL (2024). Anterior plaque brachytherapy placement for treatment of iris and iridociliary melanomas - surgical procedure and institutional experience. Indian J Ophthalmol.

[2] Lomi N, Chawla B, Das D (2024). Disinsert, retract and rotate technique of plaque brachytherapy. Indian J Ophthalmol.

[3] Lomi N, Das D, Chawla B, Herle A (2025). Inferior oblique sparing brachytherapy plaque placement for juxtafoveal peripapillary choroidal melanoma. Indian J Ophthalmol.

[4] Chaudhry IA, Liu M, Shamsi FA, Arat YO, Shetlar DJ, Boniuk M (2009). Corneoscleral necrosis after episcleral Au-198 brachytherapy of uveal melanoma. Retina.

[5] Finger PT (2001). Plaque radiation therapy for malignant melanoma of the iris and ciliary body. Am J Ophthalmol.

[6] Marinkovic M, Horeweg N, Laman MS (2018). Ruthenium-106 brachytherapy for iris and iridociliary melanomas. Br J Ophthalmol.

[7] Semenova E, Finger PT (2013). Amniotic membrane corneal buffering during plaque radiation therapy for anterior uveal melanoma. Ophthalmic Surg Lasers Imaging Retina.

[8] Sharma N, Sah R, Priyadarshini K, Titiyal JS (2023). Contact lenses for the treatment of ocular surface diseases. Indian J Ophthalmol.

